# Mesenchymal/non-epithelial mimickers of neuroendocrine neoplasms with a focus on fusion gene-associated and SWI/SNF-deficient tumors

**DOI:** 10.1007/s00428-021-03156-9

**Published:** 2021-08-05

**Authors:** Atsuko Kasajima, Björn Konukiewitz, Anna Melissa Schlitter, Wilko Weichert, Jan Hinrich Bräsen, Abbas Agaimy, Günter Klöppel

**Affiliations:** 1 Department of Pathology, Technical University Munich, Trogerstr. 18, 81675 Munich, Germany; 2 The German Cancer Consortium (DKTK), Heidelberg, Germany; 3 Department of Pathology, Universitätsklinikum Schleswig-Holstein, Campus Kiel, Christian-Albrechts-Universität zu Kiel, Kiel, Germany; 4 Institute of Pathology, Hannover Medical School, Hannover, Germany; 5 Institute of Pathology, Friedrich-Alexander-University, Erlangen-Nürnberg, University Hospital, Erlangen, Germany

**Keywords:** Neuroendocrine neoplasms, Mimics, Mesenchymal neoplasms, Genetic features

## Abstract

Mimickers of neuroendocrine neoplasms (NEN) include a number of important pitfall tumors. Here, we describe our experience with mesenchymal mimics of NENs to illustrate their spectrum and draw the attention particularly to a group of mesenchymal/non-epithelial neoplasms (MN) that combine epithelioid histology with neuroendocrine (NE-) features and peculiar genetic abnormalities. In a consultation series of 4498 cases collected between 2009 and 2021, 2099 neoplasms expressing synaptophysin and/or chromograninA were reviewed and analyzed. A total of 364 (18%) were diagnosed as non-NENs, while the remaining tumors were NEN. The group of mesenchymal/non-epithelial neoplasms with NE-features (MN-NE) included 31/364 (8%) cases. These mostly malignant neoplasms showed an epithelioid morphology. While all but one tumor expressed synaptophysin, mostly patchy, only 10/29 (34%) co-expressed chromograninA. A total of 13/31 (42%) of the MN-NE showed *EWSR1*-related gene fusions (6 Ewing sarcomas, 5 clear cell sarcomas, and 1 desmoplastic small round cell tumor, 1 neoplasm with *FUS-CREM* gene fusion) and 7 (23%) were SWI/SNF (SMARCB1 or SMARCA4)-deficient neoplasms. The remaining MN-NE included synovial sarcoma, sclerosing epithelioid mesenchymal neoplasm, melanoma, alveolar soft part sarcoma, solitary fibrous tumor, and chordoma. A total of 27/31 MN-NE were from the last 8 years, and 6 of them were located in the pancreas. Eleven MN-NE were initially diagnosed as neuroendocrine carcinomas (NECs). MN-NE with epithelioid features play an increasing role as mimickers of NECs. They mostly belong to tumors with gene fusions involving the *EWSR1* gene, or with SWI/SNF complex deficiency. Synaptophysin expression is mostly patchy and chromograninA expression is infrequent in MN-NE of this series and data extracted from literature.

**Supplementary Information:**

The online version contains supplementary material available at 10.1007/s00428-021-03156-9.

## Introduction

Neuroendocrine neoplasms (NENs) originate from various epithelial or neuroectodermal tissues. The NENs are defined by the expression of the neuroendocrine (NE) markers synaptophysin and chromograninA [27, 28]. However, these markers, either alone or both, may also be expressed in neoplasms that are not considered NENs such as complex adenocarcinomas, adrenocortical carcinomas, or sarcomas, which can therefore be confused with NENs.

Analyzing a large number of consultation cases which were received to clarify diagnostic problems in NENs and NEN-like tumors, we defined six groups of tumors and lesions that all expressed synaptophysin, usually together with chromograninA. The first and largest group (true NEN) encompasses all epithelial (cytokeratin positive) and ectodermal (cytokeratin negative) NENs. The second group assembles carcinomas with a NE-component (i.e., carcinomas that by definition do not fall into the mixed neuroendocrine non-neuroendocrine neoplasms: MiNEN category) originating from the digestive system, the lung, and the gynecological and urogenital organs and also contains carcinomas of unknown origin. The remaining four groups include acinar cell carcinomas, solid-pseudopapillary neoplasms (SPN), adrenocortical neoplasms, and mesenchymal neoplasms, all of them with NE-features. Many of the collected neoplasms with NE-features have been discussed in the differential diagnosis section of textbooks or review articles and are therefore not presented in detail.

Here, we would like to draw the attention to the group of mesenchymal/non-epithelial neoplasms with NE features, as the group includes more and more NEN-mimickers, which belong to epithelioid mesenchymal tumors with special genetic changes. We collected 31 nonepithelial NEN mimickers and present their morphology, immunohistochemical findings, genetic abnormalities, and diagnostic features.

Since many of these neoplasms do not co-express chromograninA, but only label with synaptophysin, we reviewed the literature to clarify the information about the rate of synaptophysin and chromograninA expression in the various entities of mesenchymal/non-epithelial neoplasms.

## Materials and methods

### Tissue assembling

The Consultation Centre for Pancreatic and Endocrine Neoplasms, Technical University Munich, Germany, received consultation specimens from 4498 patients between April 2009 and April 2021. All cases were reviewed at least by two endocrine and pancreas pathology experts including AK, BK, MS, WW, AA, and GK. In a few cases, the help of tertiary consultation by other expert pathologists was sought (see acknowledgments). We analyzed specimens from 4436 patients and excluded 62 cases because of insufficient materials. In all included cases, slides and/or formalin fixed paraffin embedded (FFPE) tissue blocks were available, containing sufficient material. Cases, in which we received only slides, were included, when all staining needed for final diagnosis were available. When blocks were received, recuts were done for histological and immunohistochemical examination. In some cases, a molecular pathology analysis was added. In 2529/4436 cases (57%), the final diagnosis required the use of the NE-markers synaptophysin and/or chromograninA (Supplement Fig. 1). Both markers were examined in 1937 neoplasms or lesions, and only one marker in 592 neoplasms or lesions (synaptophysin in 480, chromograninA in 112 cases). The positivity of one marker was regarded as sufficient for the criterion of NE-differentiation (83%, 2099/2529). Among 31 mesenchymal neoplasms, 4 cases were also immunostained with INSM1. After excluding 30 non-neoplastic NE-lesions, such as islet cell aggregation or PP-islet accumulations, 2069 cases were diagnosed as NE-marker positive neoplasms. Age, sex, type of specimens, and tissue origins of these patients were extracted from the available documents (see supplemental Table 1). The most frequent tissue origin was the gastrointestinal tract (35%), followed by pancreatobilliary organs (20%) and liver (16%) (data not shown). The study was approved by our local ethic committee (Internal number: 281/19 s approved on 11.06.2019).

### Histopathological and immunohistochemical evaluation

Hematoxylin and eosin (HE) and periodic acid-Schiff (PAS) staining were done on 2-µm thick FFPE tissue sections. Immunohistochemical stainings were performed using a fully automated slide preparation system (Benchmark XT, Ventanta/Roche, AZ, USA). Details regarding the immunohistochemical stainings are given in supplemental Table 2. Immunohistochemical staining was evaluated according to the percentage of positive cells, and NE-positivity was recorded, when > 5% of tumor cells stained for at least one of the NE-markers. Furthermore, we distinguished between a diffuse and patchy expression. The expression was called diffuse when all tumor cells were strongly and evenly stained, or was called patchy when the staining of the tumor cells alternates between weak and strongly and the weakly stained cells dominated. In the cases stained with the NE-marker INSM1, weak or strong nuclear staining was regarded positive. Ten pancreatic neuroendocrine tumors (PanNETs) and the islets in the surrounding pancreas served as controls.

### Diagnostic criteria of NE-expressing neoplasms

The diagnosis of the mesenchymal neoplasms with NE-features (MN-NE) followed the criteria of the WHO classification [14]. Recent publications were also taken into account, as for instance, in the case of SMARCA4-deficient neoplasms [2], sclerosing epithelial mesenchymal neoplasms (SEMN) [11], or neoplasm with *FUS-CREM* gene fusion [6, 9, 55]. Molecular testing for the detection of gene fusions was performed using different next generation sequence (NGS) panels, as previously described [5].

Thirty-one MN-NEs were identified and separated from epithelial and ectodermal NENs (including MiNENs [13, 32]) and other groups with NE-features, including carcinomas with a NE-component (i.e., carcinomas that by definition do not fall into the MiNEN category), acinar cell carcinomas (including mixed acinar carcinomas), SPN, and adrenocortical neoplasms.

### Evaluation of the referral diagnoses

The available suspected or proposed diagnoses of the referrals that concerned MN-NE were compared with the final diagnosis. 24/31 (77%) of the referrals requested confirmation of the diagnosis and/or subtyping of the tumor. The referral diagnosis was regarded either consistent or inconsistent with the final diagnosis.

## Results

### Proportional distribution of MN-NEs among NEN mimickers seen in consultation

Among 364/2069 (18%) non-NENs with NE-marker expression, MN-NE (*N* = 31) accounted for 9%. The remaining 333 non-NENs included 139 (38%) carcinomas, 76 (21%) acinar cell carcinomas, 25 (7%) SPNs, and 93 (26%) adrenocortical neoplasms (Supplemental Fig. 2, supplemental Table 1). A total of 27/31 MN-NEs were observed between 2014 and 2021.

### Features of MN-NE

**Fig. 1:**
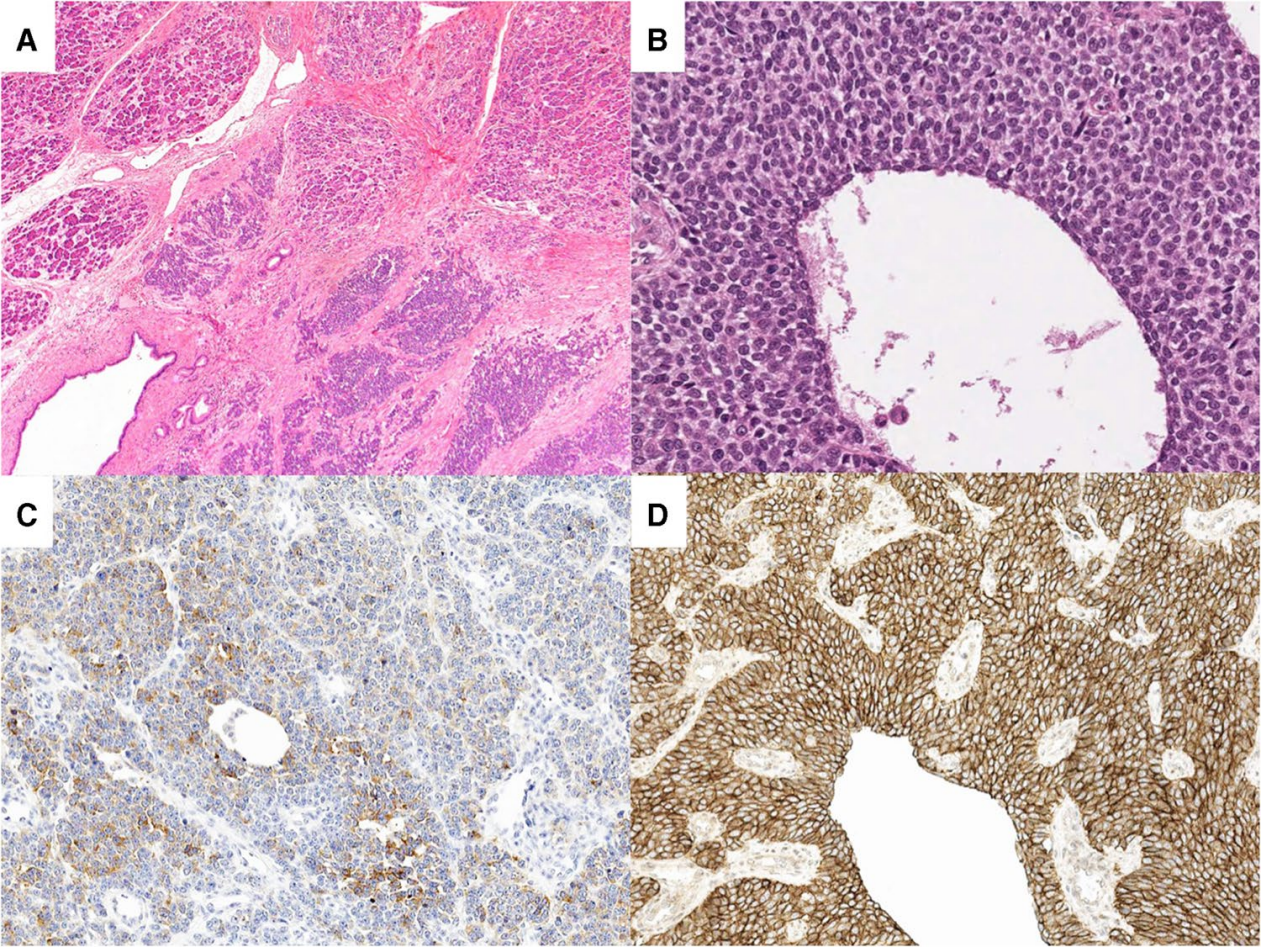
Histologic and immunohistochemical images of primary Ewing sarcoma of the pancreas with neuroendocrine features. **A** Nested cell groups embedded in sclerotic stroma infiltrating pancreatic tissue. **B** Solid and cystic growth of monomorphic round cells (hematoxylin and eosin staining). Immunohistochemical expression of (**C**) synaptophysin and (**D**) CD99

**Fig. 2:**
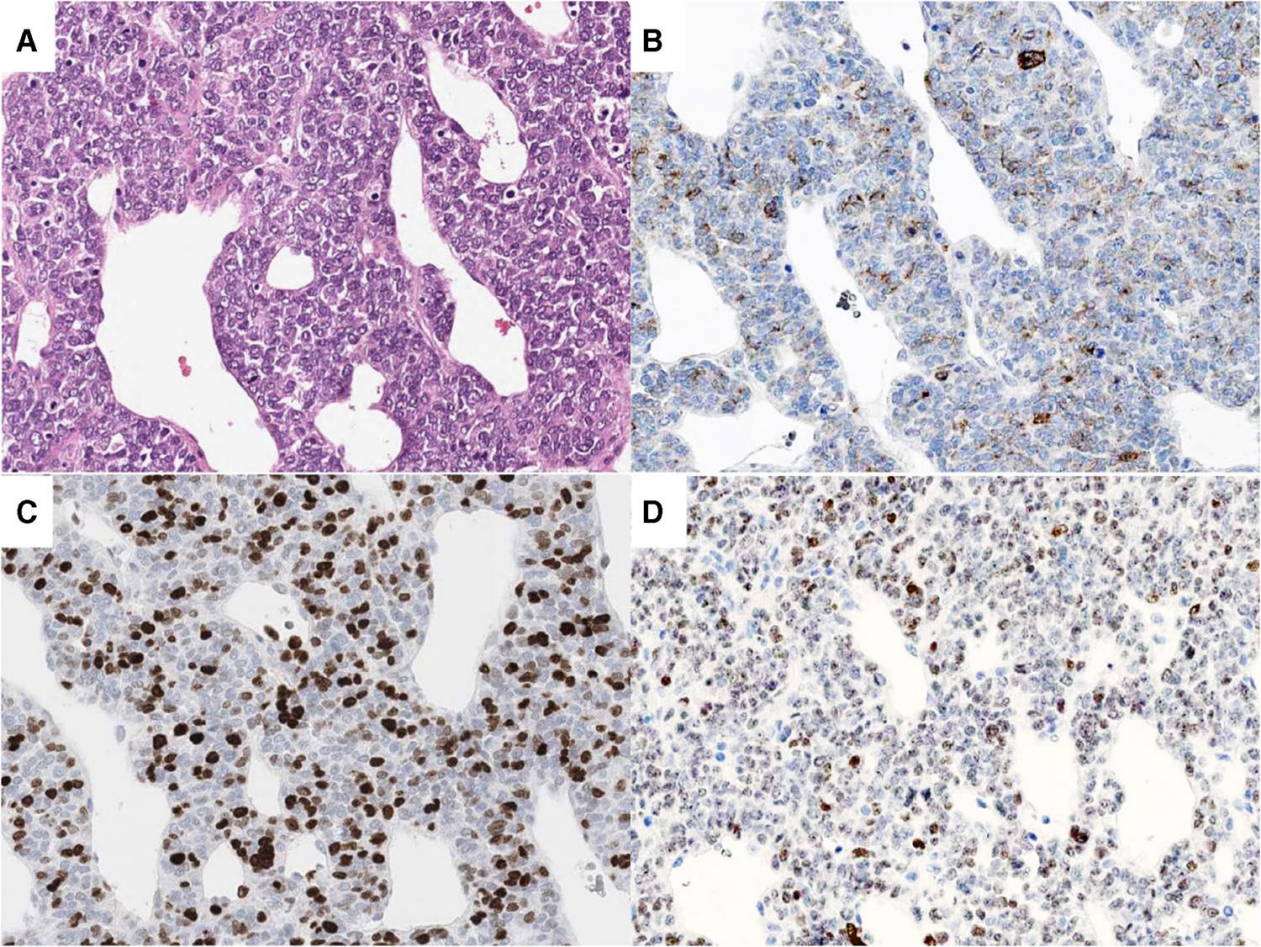
Histologic and immunohistochemical images of malignant solitary fibrous tumor with neuroendocrine features. **A** Solid growth composed of middle sized epithelioid cells and containing numerous branching vessels (hematoxylin and eosin staining). Immunohistochemical expression of (**B**) synaptophysin, (**C**) Ki67, and (**D**) STAT6

**Fig. 3:**
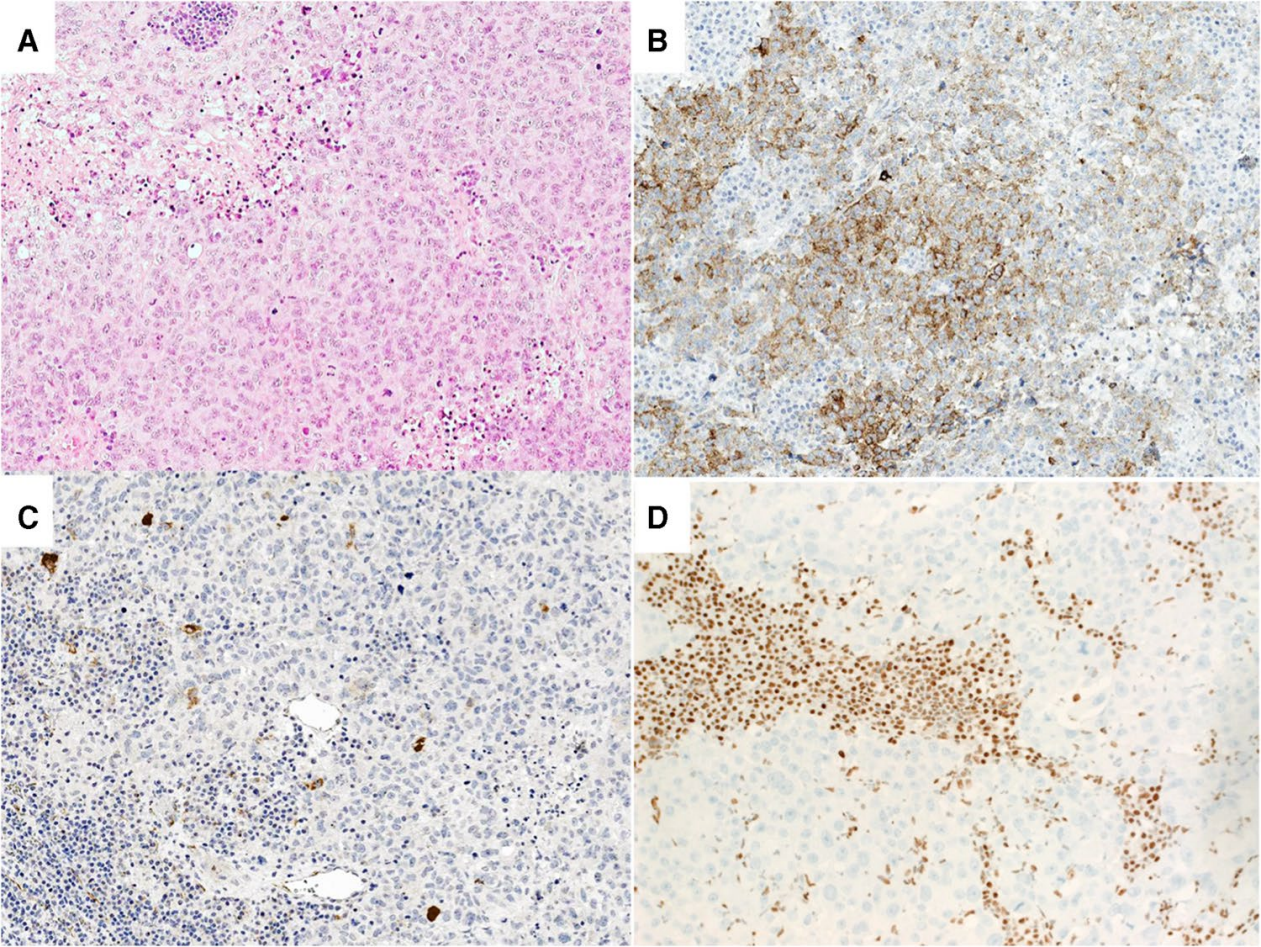
Histologic and immunohistochemical images of a lymph node metastasis of a SMARCA4-deficient neoplasm. **A** Solid growth of large pleomorphic cells. Patchy immunohistochemical expression of (**B**) synaptophysin and (**C**) cytokeratin 18 and (**D**) loss of SMARCA4

The 31 MN-NEs could be assigned to 13 entities. Most frequent were Ewing sarcoma (EWS) and clear cell sarcoma (CCS) of gastrointestinal tract (Table 1 and Supplemental Table 3). Nineteen tumors presented as primaries, 6 of them in the pancreas and 12 as metastases, mostly in the liver (Table 1, Supplemental Tables 1, 3). The tumors shared an epithelioid histology, variably combined with large cell and small round cell, spindle and rhabdoid, morphology (Figs. 1A, 2A, B, 3A) (for details, see Table 1).

**Table 1:** Pathological and molecular characteristics of 31 mesenchymal neoplasms with neuroendocrine features

Final diagnosis	*N* (%)	Findings in addition to epithelioid histology	Positive immunolabeling					Chromosomal translocation	Fusion gene
Total	31 (100)		Vimentin	CK18	SYN	CgA	Other markers		
Ewing sarcoma	6 (19)	Solid-nested, large and small round cell, cystic	2/2	2/3	6/6	2/6	CD99, WT1	t(11;22)(q24;q12) t(11;22)(q22;q12)	*EWSR1-FLI1* *EWSR1-ERG*
Clear cell sarcoma of gastrointestinal tract	5 (16)	Nested, monomorphic, cystic	2/2	0/3	5/5	2/5	S100	t(11;22)(q13;q12)	*EWSR1-ATF1* *EWSR1-CREB*
Desmoplastic small round cell tumor	1 (3)	Solid, small cell, desmoplastic	0/1	1/1	1/1	1/1	Desmin	t(11;22)(p13;q13)	*EWSR1-WT1*
Epithelioid neoplasm with FUS-CREM gene fusion	1 (3)	Solid, pseudotubular; spindelled, eosinophilic and clear cells	1/1	1/1	1/1	1/1	MUC1	t(10;16)(p11;p11)	*FUS-CREM *^*a*^
Synovial sarcoma	2 (6)	Solid, pseudotubular; spindelled, eosinophilic and clear cells	NA	2/2	2/2	NA	TLE1, EMA	t(X;18)(p11;q11)	*SS18-SSX1*
Alveolar soft part sarcoma	2 (6)	Solid, large polygonal cells, eosinophilic granular	1/1	0/2	1/2	2/2	TFE3, Desmin	t(2;13)(p11;q25)	*ASPSCR1-TFE3*
Solitary fibrous tumor, malignant	1 (3)	Solid, branching vessels, large and pleomorphic cells	1/1	1/1	1/1	0/1	STAT6	NA	
Epithelioid sarcoma	1 (3)	Solid, large, pleomorphic and rhabdoid cells	1/1	1/1	1/1	0/1	SMARCB1 (INI-1) loss	NA	
SMARCB1-deficient neoplasm	3 (10)	Nested, pseudoglandular, cystic, rhabdoid	1/1	1/1	3/3	1/3	SMARCB1 (INI-1) loss	NA	
SMARCA4 deficient neoplasm	3 (10)	Nested, large, pleomorphic and clear cells	1/1	2/2	3/3	0/3	SMARCA4 loss	NA	
Melanoma	3 (10)	Solid, spindle, pleomorphic	2/2	0/2	3/3	0/3	HMB45, MelanA, S100	NA	
Sclerosing epithelioid mesenchymal neoplasm	2 (7)	Nested, large cell, sclerosing	2/2	2/2	2/2	2/2	ERG, CD34	NA	
Chordoma	1 (3)	Nested, monomorphic, spindle, and clear cells	NA	0/1	1/1	0/1	Brachyury	NA	

*CK* cytokeratin, *SYN* synaptophysin, *CgA* chromogranin A, *NA* not analyzed

^a^NGS revealed no other mutations than SMO, which has not been yet associated with a driver gene function

All MN-NEs expressed synaptophysin (patchy in 30/31 cases) (Fig. 1C, 2B, 3B), while chromograninA, vimentin, and cytokeratin (Fig. 3C) were only expressed in 11/29 (38%, patchy 9/11), and 15/16 (94%, patchy 2/15) and 16/24 (67%, patchy 8/16) examined cases, respectively (Table 1). Four (including 1 solitary fibrous tumor; SFT, 2 SEMNs and 1 neoplasm with *FUS-CREM* gene fusion) expressed INSM1.

Ki67 index varied from 1 to 70% (Fig. 2C). High proliferation (> 40%) was observed in melanoma, epithelioid sarcoma (ES), malignant SFT, and SMARCB1- and SMARCA4-deficient neoplasms. Moderate proliferation (10–40%) was observed in EWS, CCS, synovial sarcoma (SS), and desmoplastic small round cell tumor (DSRCT) and low proliferation (< 10%) in chordoma, alveolar soft part sarcoma (ASPS), and SEMN.

Fourteen MN-NEs were diagnosed using the respective immunohistochemical key markers (Table 1, Fig. 1D, 2D, 3D). Seventeen tumors required molecular testing for the detection of gene fusions. This revealed fusions involving the Ewing Sarcoma Breakpoint Region 1 (*EWSR1*) on 22q in 12 cases. Together with their immunoprofile, these tumors were diagnosed as EWS (6 cases), CCS (5 cases), and DSRST (one case). Different gene fusions were identified in 5 neoplasms. One case had a *FUS-CREM* gene fusion [6, 9], two cases had *SS18- SSX* gene fusions characterizing SS, and another two cases had *TFE3*-*ASPSCR1* gene fusions characterizing ASPS (for details, see Table 1).

### Comparison of referral opinion/diagnosis and final diagnosis

Referral and final diagnoses were concordant in only in 2/24 (8%) cases. Diagnoses regarded as discordant were neuroendocrine carcinoma (NEC, *N* = 11), followed by NET (*N* = 3) (for details, see Supplemental Table 3). In neoplasms with small round cell morphology, such as EWS or DSRCT, small cell NEC was suggested. In neoplasms with pleomorphic and/or rhabdoid morphology such as SMARCB1-deficient neoplasms, SMARCA4-deficient neoplasms, malignant SFT, and CCS, a diagnosis of large cell NEC was proposed, while a NET diagnosis was discussed in the chordoma case and two of the CCSs.

## Discussion

Mimickers of NEN mainly represent epithelial neoplasms and they are found among various types of adenocarcinomas, acinar cell carcinomas, SPNs, and adrenocortical neoplasms. All these tumors have in common the expression of NE-markers, such as synaptophysin and chromograninA, and an epithelial nature. MN-NEs are rare, but in recent years, we observed an increasing number of these lesions, which often caused considerable diagnostic problems. In this study, we discuss in detail 31 MN-NEs, which accounted for 9% in a consultation cohort of 364 non-NEN cases (see Supplement Fig. 1).

The MN-NEs, which were spread over 13 entities of sarcomas/mesenchymal neoplasms, shared an epithelioid morphology, with spindle cell, rhabdoid, or small round cell features (Table 1). The precise diagnosis in each case was established by the expression of key immunohistochemical markers, and if needed, by molecular testing (for details, see Table 1). As key NEN markers, we used synaptophysin and chromograninA, as recommended in the recent WHO classification [27]. Other markers, such as DAXX/ATRX, alternative lengthening of telomeres (ALT), p53, and Rb1, whose abnormal expression or signaling can be helpful in classifying NETs and NECs and determining their prognosis, have not been applied to this series of tumors [25], because they are not helpful in the general distinction between NENs and non-NENs. In 6 EWSs (three of primary pancreatic origin), 5 CCSs, and one DSRST, we identified gene fusions involving the *EWSR1*. An involvement of *EWSR1* gene is probably not a specific event in ME-NEs, since its fusion with *BEND2* and *FLI1* were detected in 2 cases and 1 case, respectively, of PanNETs in a study of 102 tumors based on whole-genome sequencing [48], suggesting that histological and immunohistochemical determination has an important role in the diagnostic categorization of ME-NEs. Five neoplasms had different gene fusions including a neoplasm with *FUS-CREM* gene fusion [6, 9], two SS with *SS18- SSX* gene fusion, two ASPS with *TFE3*-*ASPSCR1* gene fusion, and one SFT with immunolabeling for STAT6 indicating a *STAT6-NAB2* gene fusion (for details, see Table 1). Another interesting group encompassing 7 neoplasms were members of the switch/sucrose non-fermenting (SWI/SNF) complex-deficient group, whose prototype is the ES. The diagnosis of these tumors was based on the immunohistochemical loss of SMARCB1- [1] or SMARCA4 [4, 46] (see Table 1).

Thirty of 31 MN-NEs were positive for synaptophysin and 11/30 (33%) cases co-expressed chromograninA. One tumor, an ASPS, expressed only chromograninA. In all the tumors, except two, the expression of both markers was patchy. We also tested selected cases with the new marker INSM1, which is thought to be a crucial regulator of neuroendocrine differentiation [22]. In the four tested cases, INSM1 labeled many or most nuclei, a pattern that was also observed in PanNETs, which served as controls.

Evaluation of our synaptophysin results with those reported in the literature in sarcomas/mesenchymal neoplasms (see Table 2) showed that synaptophysin is most frequently expressed in CCS [15, 24, 51], DSRCT [38, 41], ES [23], and neoplasms deficient for SMARCB1 [3, 12, 18] and SMARCA4 [2, 4, 44, 46, 54]. In these neoplasms, synaptophysin was recorded in approximately half to two-thirds of the cases, making synaptophysin an important “pitfall marker” in these neoplasms. Synaptophysin expression was also relatively common in EWS (on average 23%) [34, 40, 45] and melanoma (on average 35%) [47, 50], while in SFT [19, 30, 33], chordoma [39], and the pancreatic SEMN [11], synaptophysin expression seems to be rare. A new finding is the synaptophysin positivity in one of our two ASPS [16, 17, 20, 21, 26, 36, 53] and the epithelioid mesenchymal neoplasm with *FUS-CREM* gene fusion [6, 9, 35]. In contrast to the other MN-NEs with their patchy staining, synaptophysin (as well as chromograninA) labeled the *FUS-CREM* neoplasm diffusely [6].

**Table 2:** Neuroendocrine expression in mesenchymal neoplasms reported in previous studies

Entitiy	SYN	CgA	Co-expression		Location
			All cases	CgA/SYN	
	% (number positive/examined)				
Clear cell sarcoma of gastrointestinal tract ^24,15,51^	44 (14/32)	NA	NA	NA	Soft tissue
	41 (7/17)	0 (0/15)	0 (0/15)	0/7	Gastrointestinal tract
	56 (9/16)	NA	NA	NA	Gastrointestinal tract
Ewing sarcoma ^40, 45, 34^	15 (4/27)	0 (0/27)	0 (0/27)	0/4	unspecified
	25 (4/16)	NA	NA	NA	Mostly soft tissue
	8 (5/14)	8 (1/12)*	0 (0/11)	0/3	Pancreas
Desmoplastic small round cell tumor ^41,38^	16 (3/19)	5 (1/22)	Unspecified	NA	unspecified
	100 (6/6)	25 (1/4)*	0 (0/4)	0/0	Mostly abdominal cavity
Synovial sarcoma ^40,49^	0 (0/23)	0 (0/23)	0 (0/23)	0/0	unspecified
	50 (1/2)	0 (0/2)	0 (0/2)	0/1	Soft tissue/retroperitoneum
Alveolar soft part sarcoma ^36,53,26,16,16,31,20^	0 (0/7)	NA	NA	NA	Soft tissue
	0 (0/5)	0 (0/4)	0 (0/4)	0/0	Case reports: soft tissue, head and neck, prostate, mediastinum
Solitary fibrous tumor ^30,33,19^	17 (4/23)	0 (0/23)	0 (0/23)	0/4	Central nerve system
	0 (0/28)	0 (0/28)	0 (0/28)	0/0	Mostly extrapleural soft tissue
	0 (0/13)	0 (0/13)	0 (0/13)	0/0	Pleura
Epithelioid sarcoma ^23^	60 (12/20)	0 (0/20)	0 (0/20)	0/12	Soft tissue
SMARCB1 deficient neoplasm ^18,12,3^	66 (12/18)	0 (0/18)	0 (0/18)	0/12	Soft tissue
	63 (5/8)	0 (0/9)	0 (0/8)	0/5	Sinonasal tract
	18 (6/33)	10 (3/30)	4 (1/26)**	1/6	Sinonasal tract
SMARCA4 deficient neoplasm ^4,46,44,54,1^	90 (9/10)	40 (4/10)	40 (4/10)***	4/9	Sinonasal tract
	73 (16/22)	NA	NA	NA	Thoracic cavity
	18 (3/17)	0 (0/17)	0 (0/17)	0/3	Thoracic cavity
	25 (1/4)	0 (0/3)	not specified	0/1	Thoracic cavity
	12 (2/16)	0 (0/16)	0 (0/16)	0/2	Lung
Melanoma ^50,47^	45 (9/19)	0 (0/19)	0 (0/19)	0/9	Mostly metastasis
	29 (10/34)	0 (0/32)	0 (0/32)	0/10	Mostly metastasis
Sclerosing epithelioid mesenchymal neoplasm ^11^	13 (1/8)	0 (0/8)	0 (0/8)	0/1	Pancreas
Chordoma ^39^	9 (3/33)	0 (0/32)	0 (0/32)	0/3	Mostly lumbosacral
Total	32 (155/490)	2 (7/387)	1 (5/356)	5 (5/92)	

Abbreviation: *SYN* synaptophysin, *CgA* chromograninA, *NA* not analyzed

^*^ChromograninA expression only in 1 case

^**^Focal expression of both markers

^***^3 cases with focal expression of both markers, one case with diffuse synaptophysin and focal chromogranin expression

Regarding the chromograninA labeling, our data as well as the data from literature revealed a striking dichotomy of synaptophysin and chromograninA expression. Synaptophysin-chromograninA co-expression was observed in 34% of our MN-NEs and in 5/92 (5%) neoplasms from the literature [2–4, 12, 15, 18, 19, 23, 30, 33, 34, 36, 38–42, 44, 46, 47, 49, 50, 54] (see Table 1 and 2), suggesting that the dichotomy in the synaptophysin-chromograninA expression is a common finding in these neoplasms and contrasts sharply with the expression rates in PanNETs, in which chromograninA labels 91% of the synaptophysin positive tumors [52]. Among our chromograninA, negative MN-NEs were SFT, ES, SMARCA4-deficient neoplasm, melanoma, and chordoma. The literature review confirms SFT, ES, melanoma, and chordoma as tumors in which chromograninA is not or only rarely found, and adds CCS, SS, ASS, and SEMN to this list. This list can be further expanded by the adrenocortical neoplasms [31] and pancreatic SPNs [29], which are other examples of NEN mimickers characterized by chromograninA negativity in the presence of synaptophysin. The reason for the sole expression of synaptophysin is not clear, since both, synaptophysin and chromograninA, appear to be involved in the NE program that is governed by the NE-differentiation regulator INSM1, a transcription factor regulated by the Notch1-Hes1 signaling pathway [22]. However, the fact that chromograninA, as a protein which is an integral part of the neurosecretory (hormone) granule membrane, is probably only expressed when secretory granules are formed, suggests that the formation of secretory granules as a sign of complete NE-differentiation of a cell is lost earlier than the production of synaptophysin which is a constituent of synaptic-like vesicles, the function of which is not known in NE-cells but might be a more basic NE-differentiation component. In the light of these considerations, the sole expression of chromograninA, as seen in one of our two ASS and in two cases in Table 2, is difficult to understand, but might be due to a kind of protein mimicry which gives rise to unspecific immunolabeling.

Probably due to their extreme rarity, our series includes no cases of gastrointestinal glomus tumors, which were frequently found to express synaptophysin but not chromograninA [37]. We also did not observe any alveolar rhabdomyosarcoma, which may express synaptophysin and chromograninA [10]. The recently described *GLI1*-altered (*GLI1*-rearranged or amplified) malignant epithelioid soft tissue neoplasms that may show a striking neuroendocrine-like structure were not observed in our consultation series [7, 8, 43].

In 11/ 31 MN-NEs, the referral diagnosis was NECs, probably not only because they were synaptophysin and chromograninA positive, but also because some of them presented as pancreatic primaries (see Table 1). To distinguish between MN-NEs and NECs, it is important to observe the heterogeneous histology of MN-NEs in combination with the patchy expression of synaptophysin and the absent or patchy chromograninA labeling. In our MN-NEs, epithelioid cells were often mixed with spindled, small, pleomorphic, rhabdoid, or clear cells. In addition, occasionally, the epithelioid cells formed focal pseudotrabecular/pseudoglandular structures. Finally, some MN-NEs such as DSRCT, SEMN [11], and chordoma displayed a conspicuous stromal component. Such a mixture of heterologous elements is rare or absent in NECs. The other feature of MN-NEs, the patchy expression of synaptophysin and the absence or focality of chromograninA staining, are also rare in NECs, and particularly in NETs, which instead show a diffuse and usually intense staining. The complete absence of chromograninA should always arouse suspicion against the diagnosis of NEC/NET, as was seen in the chordoma case, which ran for years under the diagnosis of a metastasized NET based on a positive synaptophysin staining only, never accompanied by any chromograninA labeling. In cases of doubtful synaptophysin staining, the new NE-marker INSM1 could help clarify NE-differentiation [52]. Finally, NECs and especially NETs are diffusely cytokeratin 18-positive and usually vimentin-negative, while in MN-NE positivity for vimentin is stronger than that for cytokeratin.

**Fig. 4:**
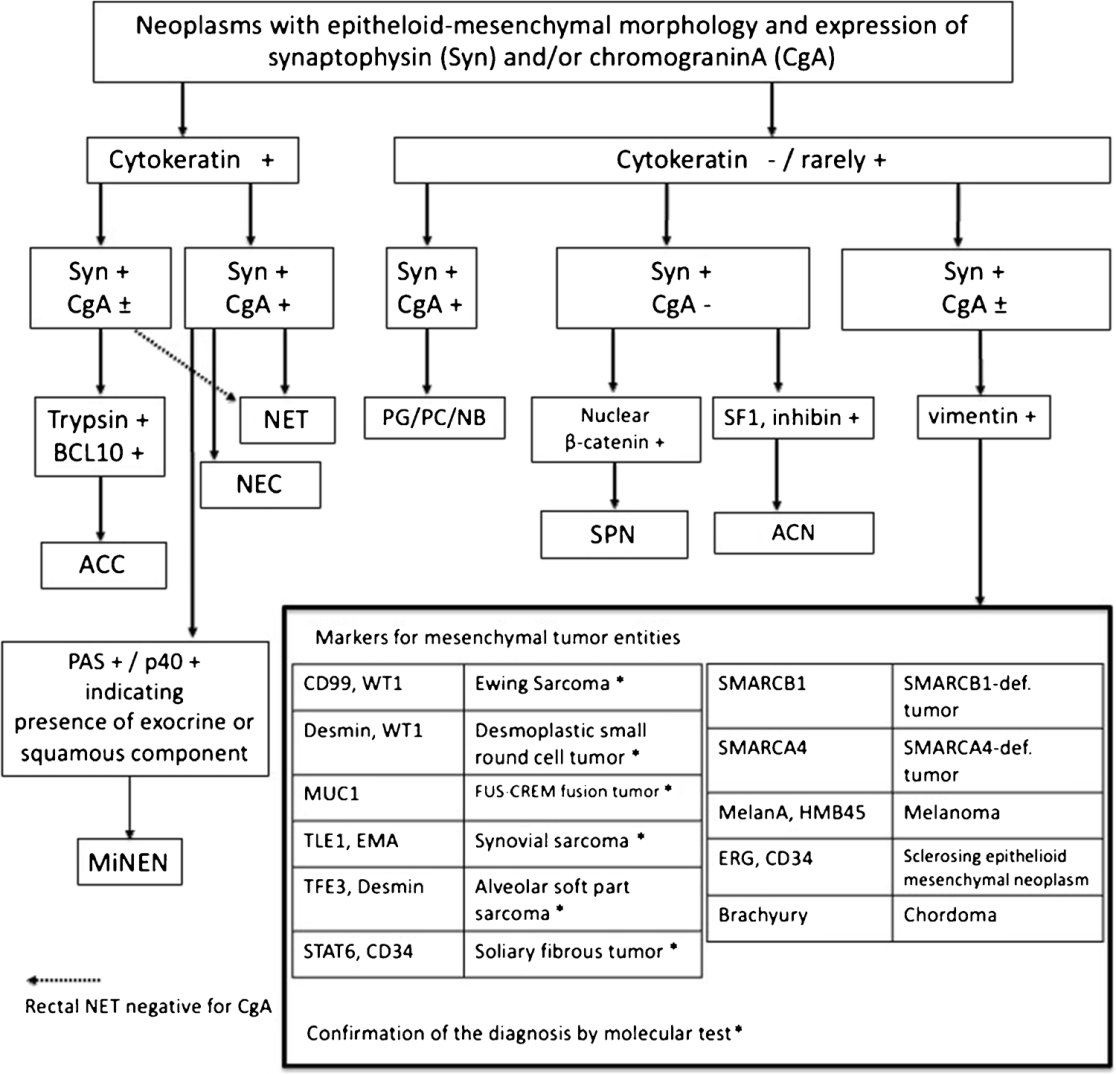
Diagnostic algorithm of tumors with neoplasms with epithelioid-mesenchymal morphology and expression of synaptophysin and/or chromograninA. Footnote: Abbreviations: NET neuroendocrine tumor, NEC neuroendocrine carcinoma, PG garaganglioma, PC pheochromocytoma, NB neuroblastoma, ACC acinar cell carcinoma, MiNEN mixed neuroendocrine nonneuroendocrine neoplasm, SPN solid-pseudopapillary neoplasm, ACN adrenocortical neoplasm, SYN synaptophysin, CgA chromograninA, SF1 seteroid factor 1

In conclusion, MN-NEs represent only a small group among the various NEN mimickers, but have been increasingly noticed in recent years and are particularly found among new entities of mesenchymal tumors that share an epithelioid-mesenchymal morphology, show a variegated immunophenotype, are characterized by gene fusion alterations in the *CREB* family or mutations of *SMARC* genes, and can occur as pancreatic primaries. All these tumors may cause diagnostic problems in the distinction from NECs and to a minor degree also NETs. However, careful analysis of morphology and immunophenotype in combination with a molecular examination usually reveals the right diagnosis (Fig. 4). The genetic mechanisms that cause and underlie the production of synaptophysin or chromograninA in non-NENs are so far not understood, but it seems that synaptophysin or synaptophysin-like proteins are more commonly produced in cells of non-NEN than chromograninA.

## Supplementary Information

Below is the link to the electronic supplementary material.

Supplemental Figure 1: Algorithm of the evaluation of 4498 consultation specimens for identification of neuroendocrine marker positive mesenchymal neoplasms. Abbreviations: NE neuroendocrine, NEN neuroendocrine neoplasm. (JPG 209 KB)

Supplemental Figure 2: Proportional distribution of neuroendocrine marker expressing non-neuroendocrine neoplasms (N=364). (JPG 99 KB)

Supplementary Tables 1-3 (XLSX 24 KB)
