# Supplementary figures and images for: Mesenchymal/non-epithelial mimickers of neuroendocrine neoplasms with a focus on fusion gene-associated and SWI/SNF-deficient tumors
Source: Virchows Arch. 2021 Aug 5;479(6):1209–19. doi: 10.1007/s00428-021-03156-9 (PMC8724147; doi:10.1007/s00428-021-03156-9)

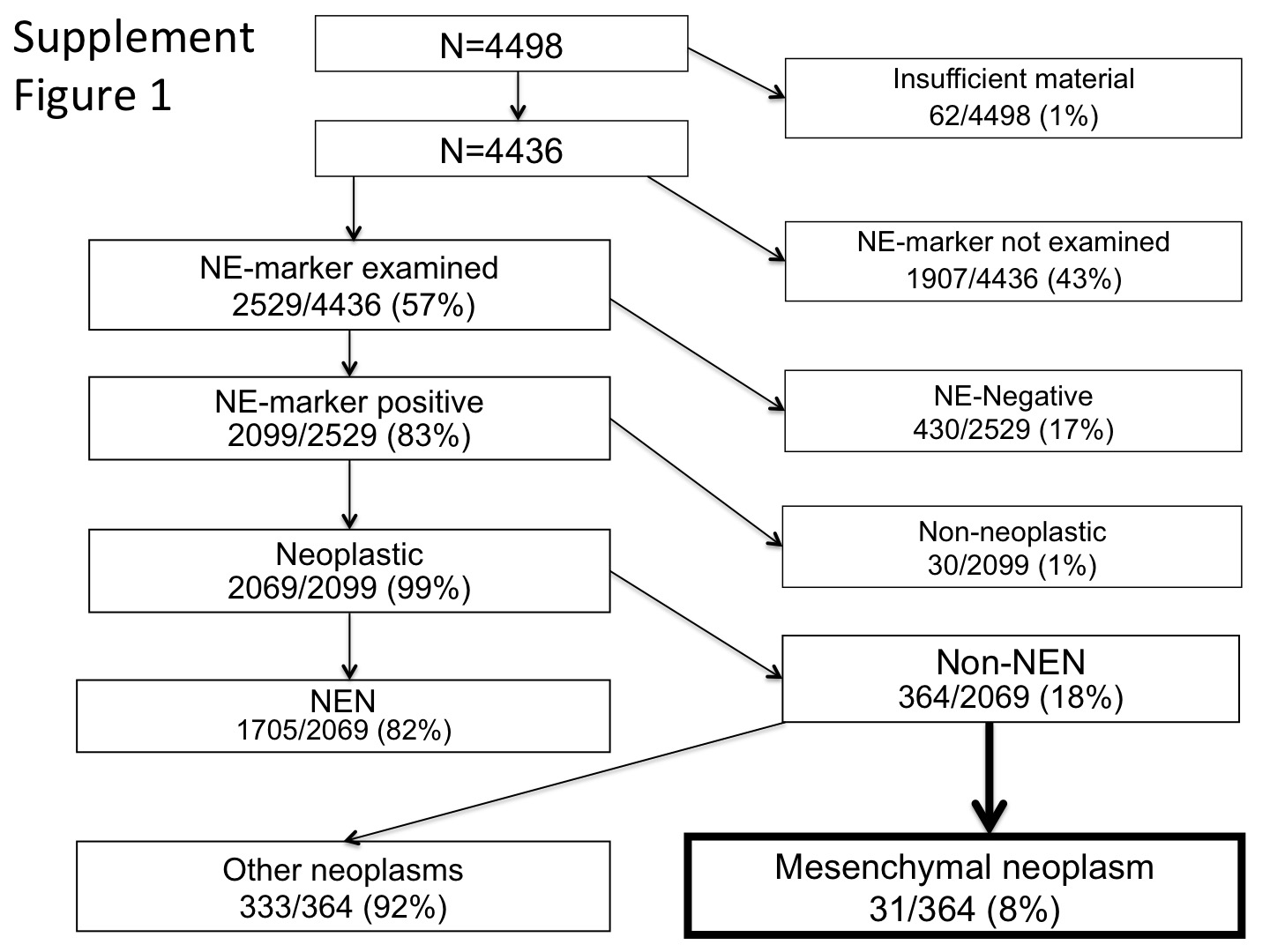

Supplement: Supplementary file 1 — Supplemental Figure 1: Algorithm of the evaluation of 4498 consultation specimens for identification of neuroendocrine marker positive mesenchymal neoplasms. Abbreviations: NE neuroendocrine, NEN neuroendocrine neoplasm. (JPG 209 KB) [file 428_2021_3156_MOESM1_ESM.jpg]

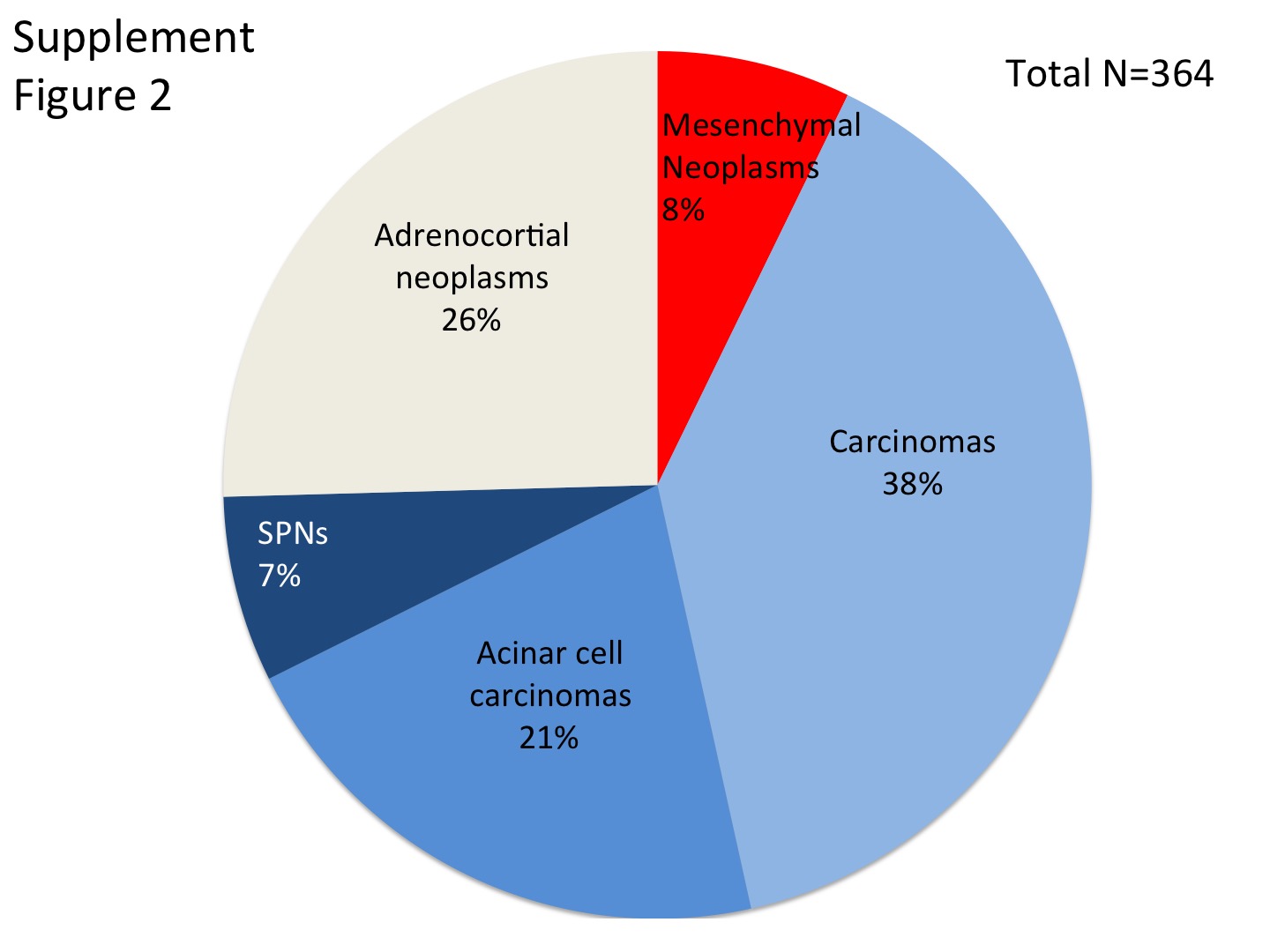

Supplement: Supplementary file 2 — Supplemental Figure 2: Proportional distribution of neuroendocrine marker expressing non-neuroendocrine neoplasms (N=364). (JPG 99 KB) [file 428_2021_3156_MOESM2_ESM.jpg]
